# A method for reconstructing temporal changes in vegetation functional trait composition using Holocene pollen assemblages

**DOI:** 10.1371/journal.pone.0216698

**Published:** 2019-05-29

**Authors:** Fabio Carvalho, Kerry A. Brown, Martyn P. Waller, M. Jane Bunting, Arnoud Boom, Melanie J. Leng

**Affiliations:** 1 Department of Geography and Geology, Kingston University London, Kingston upon Thames, United Kingdom; 2 School of Environmental Sciences, University of Hull, Hull, United Kingdom; 3 School of Geography, Geology and the Environment, University of Leicester, Leicester, United Kingdom; 4 NERC Isotope Geosciences Laboratory, British Geological Survey, Nottingham, United Kingdom; 5 Centre for Environmental Geochemistry, School of Biosciences, University of Nottingham, Loughborough, United Kingdom; Centre National de la Recherche Scientifique, FRANCE

## Abstract

Methods of reconstructing changes in plant traits over long time scales are needed to understand the impact of changing environmental conditions on ecosystem processes and services. Although Holocene pollen have been extensively used to provide records of vegetation history, few studies have adopted a functional trait approach that is pertinent to changes in ecosystem processes. Here, for woody and herbaceous fen peatland communities, we use modern pollen and vegetation data combined with pollen records from Holocene deposits to reconstruct vegetation functional dynamics. The six traits chosen (measures of leaf area-to-mass ratio and leaf nutrient content) are known to modulate species’ fitness and to vary with changes in ecosystem processes. We fitted linear mixed effects models between community weighted mean (CWM) trait values of the modern pollen and vegetation to determine whether traits assigned to pollen types could be used to reconstruct traits found in the vegetation from pollen assemblages. We used relative pollen productivity (RPP) correction factors in an attempt to improve this relationship. For traits showing the best fit between modern pollen and vegetation, we applied the model to dated Holocene pollen sequences from Fenland and Romney Marsh in eastern and southern England and reconstructed temporal changes in trait composition. RPP adjustment did not improve the linear relationship between modern pollen and vegetation. Leaf nutrient traits (leaf C and N) were generally more predictable from pollen data than mass-area traits. We show that inferences about biomass accumulation and decomposition rates can be made using Holocene trait reconstructions. While it is possible to reconstruct community-level trends for some leaf traits from pollen assemblages preserved in sedimentary archives in wetlands, we show the importance of testing methods in modern systems first and encourage further development of this approach to address issues concerning the pollen-plant abundance relationship and pollen source area.

## Introduction

Recent studies have highlighted the importance of gaining a long-term perspective on the response of ecosystems to environmental change [1, 2]. Such a perspective is crucial to help parameterise numerical models, which would improve ecological predictions and help develop better-informed conservation strategies. However, many questions remain unresolved on how to reconstruct long-term changes in ecosystems. Palaeoecological information, particularly Holocene pollen data, may be the crucial link in such reconstructions [3]. Although palaeoecological investigations have been extensively used to provide records of past plant associations and vegetation and land use history (e.g., [4–8]), there have been comparatively few palaeoecological studies using plant functional traits that are pertinent to changes in ecosystem processes (but see Lacourse [9] and reviews in Birks [10] and Birks et al. [11]).

Differences between plant traits may play an important role in the long-term response of plant communities to environmental change and human impact [12]. Temporal changes in environmental conditions may interact with changes in vegetation physiology and functional traits [9] to affect the rates of delivery of important ecosystem processes through time [13]. Key plant traits (e.g., leaf structure and nutrient content) influence species’ fitness and performance [14] and affect ecosystem processes [15, 16] and services [17, 18]. Leaf nutrients and leaf area-to-mass ratios are known to control plant respiration, carbon acquisition, water transfer and other aspects of plant metabolism [19, 20]. The implications for ecosystem processes (e.g., biogeochemical cycling) are evident as leaf construction and nutrient content will regulate the amount of recalcitrant compounds (e.g., lignin) of dead litter decomposing in the soil [21–23], influencing microbial activity and organic matter mineralisation and accumulation rates [24, 25].

One of the most important challenges in reconstructing changes in plant trait composition over long time scales lies in the use of numerical techniques to link plant functional information to pollen data [10]. Some previous studies have relied on broad plant functional types to determine global biome shifts through time (e.g., [26–28]), but have not undertaken finer resolution reconstructions using functional traits pertinent to changes in ecosystem processes. Recent investigations linking pollen data to specific plant traits have advanced our understanding of Holocene vegetation functional change and showed that pollen data can generally reflect long-term trends in plant trait diversity based on different measures of functional diversity (e.g., [12, 29]). However, they either relied on complex vegetation cover modelling algorithms and simulation studies to validate their pollen-derived diversity measures or were restricted to pollen records extracted from lake sediments with a landscape-level source area. Moreover, these studies were largely concerned with the relationship between plant traits and past climate change, community assembly processes and disturbance. Analyses that explore the links between temporal changes in vegetation traits and ecosystem processes (e.g., biomass accumulation and decomposition rates) while taking advantage of simpler numerical methods that use both modern and Holocene pollen datasets are still lacking in the literature. Enhancing our ability to use palaeoecological data, particularly those collected through pollen analysis, is important given the technique’s ability to reconstruct peatland vegetation from which important ecosystem services are derived [30–32]. An additional reason for focussing on pollen is the quantity and accessibility of data available through repositories organised into common formats for many regions of the world (e.g., [33, 34]).

Fen peatlands are suitable ecosystems in which to link palaeoecological data to plant traits to reconstruct functional vegetation history, with pollen analysis offering the possibility of ecosystem-scale reconstructions in spatially heterogeneous environments. Fens occupy the seasonally and periodically flooded habitat zone between swamps and dry land and have high biodiversity [35–38] that reflects various environmental gradients (e.g., water level, pH, fertility). They formerly occurred extensively, and peat derived from such environments makes up a significant proportion of the palaeoecological resource available in lowland Europe, notably in the area surrounding the southern North Sea [39].

**Fig 1:**
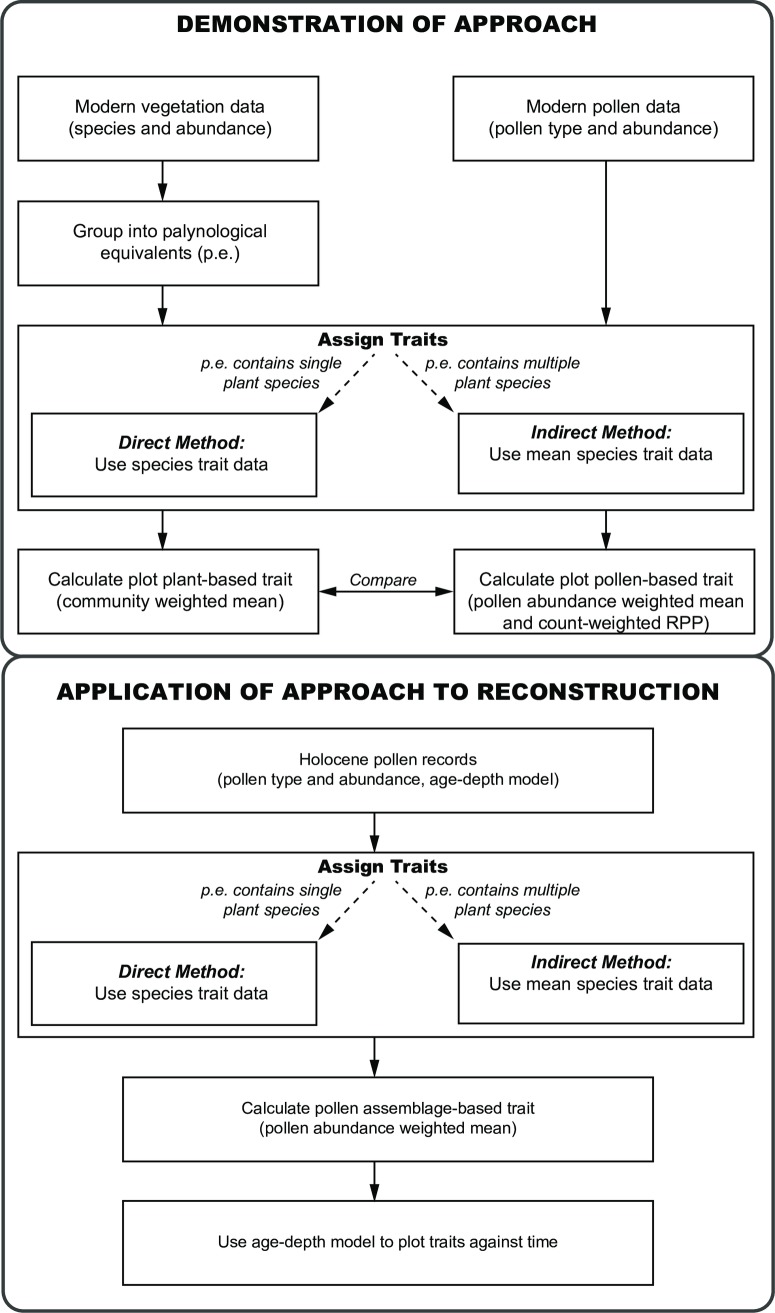
Steps involved in reconstructing long-term changes in functional traits by linking plant traits to pollen data. The modern vegetation data are firstly grouped into palynological equivalents of the modern pollen types to account for the limitations imposed by pollen taxonomic resolution. Trait values are then assigned to the modern pollen types via direct or indirect trait attribution and by weighting pollen counts by relative pollen productivity (RPP; see text). Next, modern pollen assemblages are related to the modern vegetation to establish how well the trait data of the contemporary vegetation reflect that of the modern pollen taxa to determine the extent to which pollen assemblages can be used to predict the trait composition of the vegetation. This is a vital step that has been missing from previous studies but important if extrapolating results to other sites. Pollen data from dated sediment deposits are then used to reconstruct temporal changes in trait patterns using the best fitting traits between the contemporary vegetation and the modern pollen taxa. Traits are assigned to Holocene pollen assemblages by attributing mean trait values (directly and indirectly) measured from the vegetation. Leaf trait community-weighted means (CWMs) for the Holocene pollen samples can then be calculated.

Here, we demonstrate a new method (Fig 1) for using Holocene pollen records to reconstruct the functional characteristics of past plant communities in lowland fens to better understand historical ecosystem processes in wetlands. First, we compare plot-level (≈ 12.6 m^2^) trait variation seen in the local fen vegetation with that calculated from modern pollen assemblages originating from moss polsters. We then select the traits that show the best fit between the modern pollen- and vegetation-derived measures and apply the modelled relationship to Holocene pollen assemblages to reconstruct past trait dynamics and temporal changes in functional vegetation patterns.

## Methods

### Vegetation survey and modern pollen data

Natural England and the Norfolk Wildlife Trust gave access to Woodwalton Fen (52°26’N 0°11’W) and Upton Broad (52°40’N 1°31’E), respectively and granted permission for the collection of plant and soil samples. Modern vegetation and modern pollen (trapped in the green parts of mosses) were sampled throughout the summers of 2013 and 2014 from 136 circular sampling plots (2-m radius) at Upton and Woodwalton, respectively. These two sites are located in eastern England and are comprised of both woody and herbaceous fen vegetation (S1 Fig). Vascular plants were counted on a first ‘hit’ basis for each species using a point quadrat method and moss samples were located close to the central point of the sampling plot. Details of the sampling design and a description of the sites can be found in S1 Appendix and in Waller et al. [40].

### Holocene pollen data

Holocene pollen data were derived from cores originating from two large former coastal wetlands in the UK, the East Anglian Fenland (*c*. 4,000 km^2^) and the Romney Marsh area (*c*. 250 km^2^), in which thick sequences of peat, largely of fen origin, accumulated during the mid- to late-Holocene. Eight fossil pollen sequences (five from Fenland and three from Romney Marsh) were selected for analysis. All records show the alternating development of woody (notably dominated by *Alnus* and *Betula* pollen) and herbaceous (dominated by Poaceae and Cyperaceae) fen (S2 Appendix, S1 Table) and were classified accordingly. A threshold for herbaceous/woody classification of > 30% total land pollen (TLP) from herbaceous taxa (classified as herb-dominated) and > 70% TLP from woody taxa (classified as wooded) was based on the over-representation of tree pollen in these environments [41, 42]. The pollen taxa included in the analyses were those with contemporary plant equivalents locally present at the modern sites and for which we measured functional traits.

### Leaf trait data

Six leaf traits [specific leaf area (SLA), leaf dry-matter content (LDMC), leaf C and N content (%C and %N), leaf C/N ratio and leaf δ^13^C] were selected due to their well-known links to several ecosystem processes [43–46], and measured on individual samples of vascular plants collected from Upton and Woodwalton following standardised protocols [47]. See S3 Appendix for details of leaf sampling. Sampling effort focused on the most abundant species across Upton and Woodwalton (S3 Appendix), based on the premise of the biomass ratio hypothesis that the most abundant plants are likely to have greater modulating effects on ecosystem processes [44, 45]. Trait data coverage of the modern vegetation was approximately 98% of total species cover (S3 Appendix), while modern and Holocene pollen assemblages showed approximately 93% and 99.4% trait coverage, respectively.

### Reconstructing holocene trait composition

#### Testing the approach using modern data

The approach taken to link the Holocene pollen data to plant traits is outlined in Fig 1. Firstly, we grouped the contemporary vegetation data from Upton and Woodwalton into palynological equivalents (p.e.) of the modern pollen types (S2 Table), since many pollen grains can only be identified to the genus or family levels (e.g., *Quercus*, *Salix*, Poaceae, Cyperaceae). A total of 103 modern pollen (and spore) types were identified, with 22 types (21.4%) being palynologically distinguishable at the species level (i.e., pollen types that can be directly attributed to an individual species found in the vegetation; [40], S2 Table).

Secondly, leaf trait values were assigned to the modern pollen types using two different methods. First, for palynological-equivalent taxa made up of multiple plant species that produce indistinguishable pollen and are normally grouped together as one pollen type, we indirectly attributed mean trait values of their site-level pollen-equivalent vegetation for which trait data were available (see S3 Table for trait means and standard deviations of multispecies modern pollen types). Second, for pollen types that can be identified to species level, we directly assigned trait values from the equivalent species recorded in the modern vegetation.

Thirdly, community weighted means (CWMs) of leaf traits were estimated at the sampling plot-level for both the modern pollen assemblages and the contemporary vegetation. A plot-level comparison is appropriate due to the overriding effect of the functional characteristics of the dominant vegetation on ecosystem functioning [44], which has been well supported by theory and empirical evidence [48, 49]. Plot-level CWMs were calculated by transforming species counts into relative abundances by scaling them to the total count of a given sampling plot, then using these as weighting factors for the calculation of a single mean value for each trait.

The next step, often omitted in previous studies but important when extrapolating results, was to determine if we could confidently use traits assigned to pollen assemblages to reconstruct traits found in the vegetation. We did this by fitting linear mixed effects models (LMEs) that determined the relationship between community mean trait values of the modern vegetation and the estimated mean trait values derived from the modern pollen assemblages for the individual sample points. We used relative pollen productivity (RPP) correction factors in an attempt to improve this relationship by using the ‘Standard 3’ RPP values from Mazier et al. [50], which are based on a critical analysis of all available published studies of RPP from northern Europe. RPP values were directly available from the Mazier et al. [50] dataset for 11 palynologically distinct pollen types, and a further two pollen types were assigned RPP values from closely related taxa (*Cirsium* and *Eupatorium* were assigned RPP values from *Leucanthemum*-type since they are all within the Compositae Tubuliflorae group of pollen types, while *Rumex sanguineus* was assigned the RPP value for *Rumex acetosa*). *Cladium* pollen was added to the Cyperaceae total for the analysis. These 13 p.e. types (eight herbaceous and five woody types) made up 82% of the total pollen counted from the moss samples on average, with a plot-level median value of 84% and a range of 54–94%. Two estimates of each of the six traits were then calculated for the taxa with RPP values, the count-weighted trait (*T*_*cw*_) and the adjusted count-weighted trait (*T*_*acw*_) as:

$$T_{cw}=\frac{\sum_{1}^{m}c_{i}\times t_{i}}{\sum_{1}^{m}c_{i}}$$

$$T_{acw}=\frac{\sum_{1}^{m}{R_{i}\times c}_{i}\times t_{i}}{\sum_{1}^{m}{R_{i}\times c}_{i}}$$

where *m* is the number of taxa and *c*_*i*_, *t*_*i*_ and *R*_*i*_ are the pollen count, mean trait value and RPP value of taxon *i*, respectively. The range of reconstructed trait values was substantially larger after RPP adjustment for SLA (+49% change) and LDMC (+11% change), but smaller for leaf C/N ratio (-25% change) and little changed for other traits. We also estimated the count-weighted (*T*_*cw*_) linear relationship between vegetation and modern pollen using 44 pollen types (34 herbaceous and 10 woody types) with and without RPP estimates available. These 44 types were those with contemporary plant equivalents locally present at the modern sites and for which we measured functional traits (i.e., the *T*_*cw*_ analysis using these 44 pollen types was not restricted to the 13 pollen types with RPP estimates available).

LMEs were fitted using sites as the random factor, CWMs of the contemporary fen vegetation as the dependent variable and the CWMs of the pollen samples (with and without RPP adjustment) as the fixed factor (explanatory variable). The objective was to compare expected values (vegetation traits) with pollen-inferred trait values to determine which traits can be assigned to pollen assemblages to reconstruct traits found in the vegetation (i.e., to test the predictive power of pollen-derived traits). Model parameters were estimated by maximum likelihood, while goodness-of-fit between the modern pollen and the modern vegetation was assessed by marginal *r*^2^ [51] and by root mean square error of prediction (RMSEP). RMSEP measures the predictive capacity of a model and compares predicted (fitted) values of a model with observed values (squared deviations). Therefore, the lower the RMSEP, the higher the predictive ability of the model. LMEs were fitted using the *lme* function in the *nlme* package [52] in *R* 3.5.2 [53]. Marginal *r*^2^ were calculated with the *piecewiseSEM* package [54] and RMSEP values were estimated with the *RMSEP* function in the *chillR* package [55] using the fitted values of the random effects. We performed the analyses using herbaceous and woody taxa separately and in combination. Herbaceous taxa were recorded in all 134 sampling plots while woody taxa were recorded in 72 plots.

#### Reconstructing plant trait variation over time

Once the linear relationships between modern pollen samples and the contemporary vegetation had been determined, the best fitting traits were selected as the most appropriate for the functional reconstruction of the Holocene pollen record. We assigned traits to sediment-record pollen types by using means of the trait values of the contributing taxa to that pollen type (See S4 Table for trait means and standard deviations of multispecies pollen types). We then calculated community (sample) weighted mean values of these traits and plotted them against ^14^C-ages. Chronologies were constructed for the eight Holocene sites using linear interpolation based on the radiocarbon dates of selected samples published in Waller [56] and Waller et al. [57].

In order to determine the extent to which the sampled modern fen communities differ from the reconstructed Holocene fen communities, we calculated means and 95% confidence intervals (using two-tailed Student’s *t* at *α* = 0.025) of the CWMs of leaf traits of the modern herbaceous and woody vegetation (i.e., plant-based data) to characterise average conditions expected to be found in contemporary fen communities, against which the leaf traits derived from the Holocene pollen samples were compared by means of one-observation *t*-tests [58].

## Results & interpretation

### Predicting vegetation leaf traits from modern pollen assemblages

**Fig 2:**
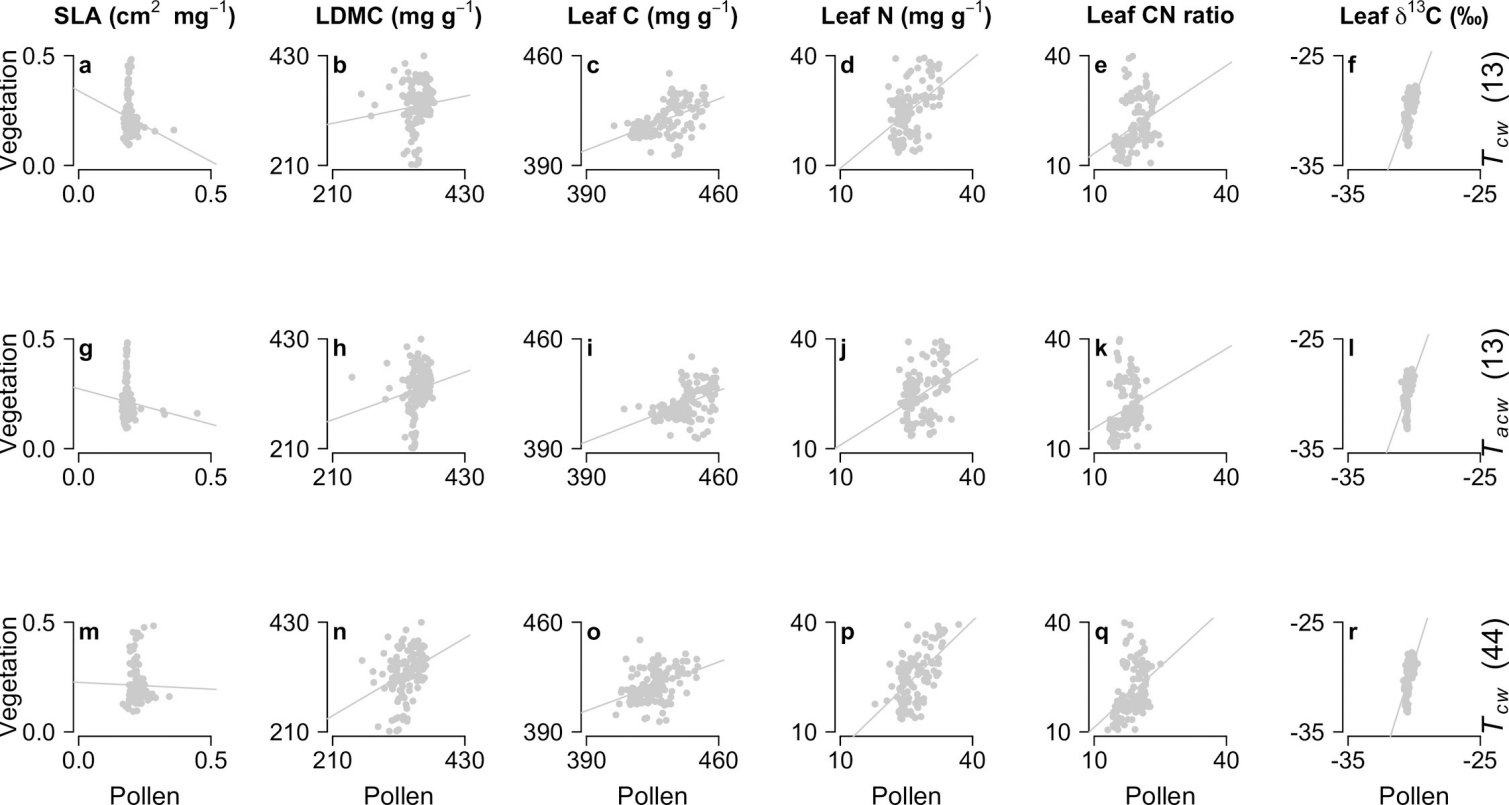
**Linear relationships between the CWMs of vegetation and modern pollen before (top row, panels a to f) and after (middle row, panels g to l) relative pollen productivity (RPP) adjustment (*T***_***cw***_ **and *T***_***acw***_**, respectively) using the 13 pollen types for which RPP estimates were available.** The bottom row (panels m to r) shows the relationship between vegetation and modern pollen including all 44 pollen types with and without RPP estimates and with trait data available. Marginal *r*^2^ and RMSEP values of linear mixed effects models (LMEs) of the standing vegetation as a function of the modern pollen data are shown in Table 1. Marginal *r*^2^ and RMSEP were used to determine how well plot-level mean trait data of the modern pollen assemblages can predict mean trait values of the modern fen vegetation.

**Fig 3:**
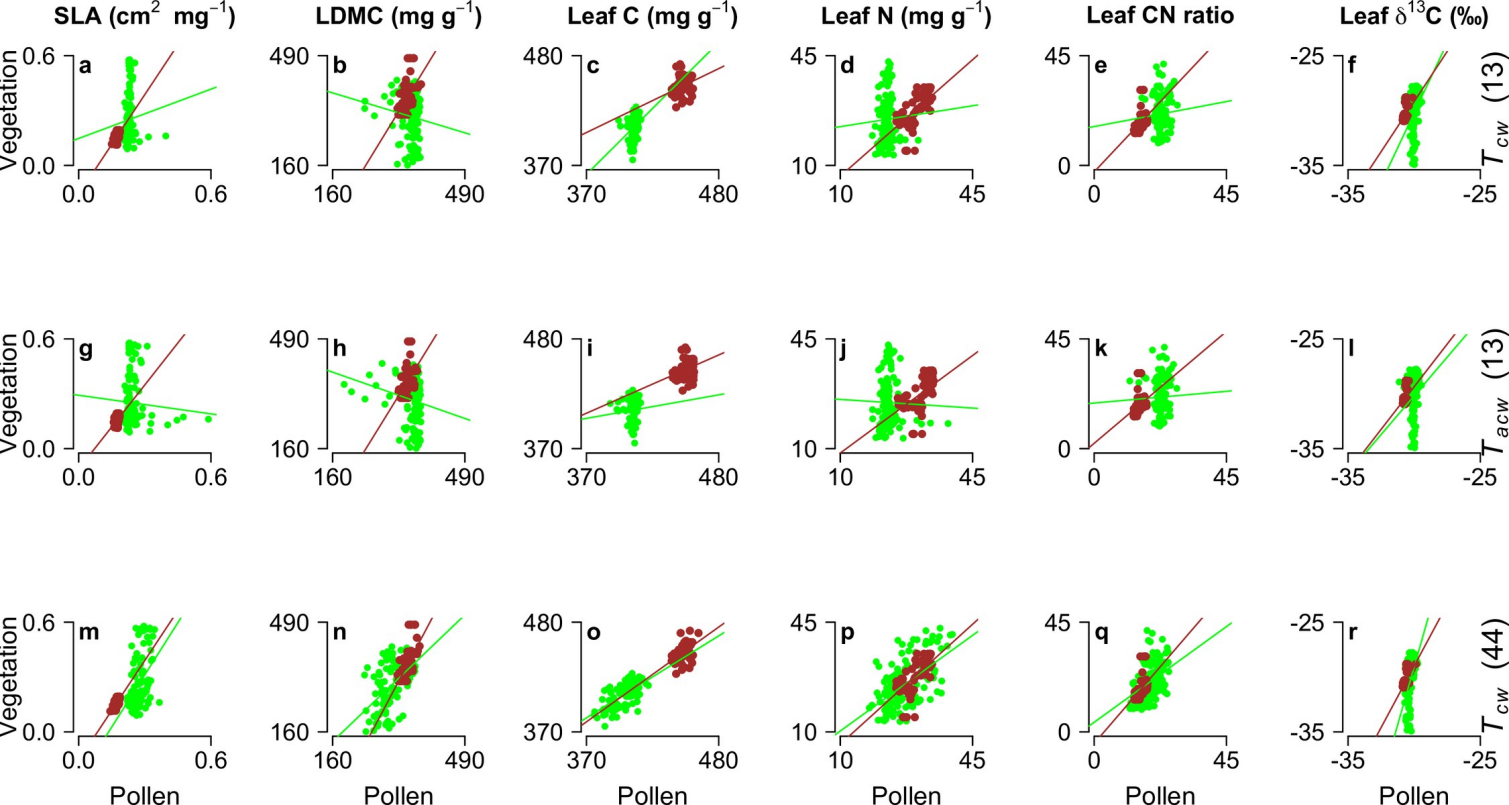
**Linear relationships between the CWMs of herbaceous (green circles) and woody (brown circles) vegetation and modern pollen before (top row, panels a to f) and after (middle row, panels g to l) relative pollen productivity (RPP) adjustment (*T***_***cw***_ **and *T***_***acw***_**, respectively) using the 13 pollen types (eight herbaceous and five woody types) for which RPP estimates were available.** The bottom row (panels m to r) shows the relationship between vegetation and modern pollen including all 44 pollen types (34 herbaceous and 10 woody types) with and without RPP estimates and with trait data available. Marginal *r*^2^ and RMSEP values of linear mixed effects models (LMEs) of the standing vegetation as a function of the modern pollen data are shown in Table 1. Marginal *r*^2^ and RMSEP were used to determine how well plot-level mean trait data of the modern pollen assemblages can predict mean trait values of the modern fen vegetation.

Overall, the CWMs of leaf traits calculated from the modern vegetation in the 136 plots showed higher variability (*y* axis in Figs 2 and 3) than the CWMs calculated from the contemporary pollen taxa (*x* axis in Figs 2 and 3). Three possible explanations for the relatively low variability of traits calculated from the modern pollen assemblages across the 136 plots can be advanced. Pollen taxa occur with different abundances than the palynological-equivalent plants, due to inter-specific differences in pollen production and dispersal. Some pollen will also originate from beyond the sampled plot, and as a result the pollen signal from the within-plot vegetation will be ‘diluted’. There may also be some reduction of variability caused by the averaging of trait values to characterise taxa with indistinguishable pollen, especially for samples with high Poaceae and Cyperaceae pollen values. Consequently, while the weighted-mean traits of the vegetation emphasise the characteristics of the dominant life forms, the pollen assemblages may smooth any extreme trait values presented by the dominant plants of the immediate vegetation.

The leaf traits based on elemental analyses (C- and N-based traits) generally showed better linear fits between the contemporary vegetation and the modern pollen (LME analyses; Figs 2 and 3 and Table 1), particularly when including the pollen types for which RPP estimates were not available (i.e., 44 pollen types; bottom rows in Figs 2 and 3 and Table 1). Marginal *r*^2^ were generally lower when considering only the 13 p.e. types with RPP estimates (top rows in Figs 2 and 3 and Table 1), and RPP adjustment did not improve the relationship between pollen and vegetation traits (i.e., *r*^2^ were generally higher for *T*_*cw*_ than *T*_*acw*_ estimates; middle rows in Figs 2 and 3 and Table 1). Herbaceous taxa commonly showed higher *r*^2^ values than all taxa in combination, particularly for SLA and LDMC, but also higher RMSEP values than when analysing all taxa in combination (Table 1). Woody taxa showed better linear fits in almost all cases, both in terms of *r*^2^ and RMSEP values (Table 1). Specific leaf area (SLA) showed relatively low *r*^2^ values between pollen and vegetation in almost all cases, while leaf dry-matter content (LDMC) showed the highest RMSEP values (Table 1). Leaf C and N content, leaf C/N ratio and leaf δ^13^C showed relatively high *r*^2^ values and low RMSEP values (Table 1). This may have been caused by the relatively high variability in the elemental leaf traits of the pollen samples compared to SLA and LDMC, reducing the residuals of the fitted linear models of the leaf elemental traits and providing a better fit to the vegetation data.

**Table 1:** *r*^2^ and RMSEP (root mean square error of prediction) values of the linear relationship between modern pollen and vegetation. *n* pollen and *n* veg columns refer to the number of pollen types and vegetation species used in the analysis with count-weighted (***T***_***cw***_) and adjusted count-weighted traits (***T***_***acw***_). *r*^2^ values in bold are significant at *p* < 0.05. SLA = specific leaf area; LDMC = leaf dry-matter content.

	*n* pollen	*n* veg.	SLA		LDMC		Leaf C		Leaf N		Leaf C/N ratio		Leaf δ^13^C	
all taxa (134 plots)			*r*^2^	RMSEP	*r*^2^	RMSEP	*r*^2^	RMSEP	*r*^2^	RMSEP	*r*^2^	RMSEP	*r*^2^	RMSEP
*T*_*cw*_	13	64	**0.04**	0.07	0.03	42.28	**0.22**	7.41	**0.22**	5.73	**0.12**	6.04	**0.4**	0.99
*T*_*acw*_	13	64	0.01	0.07	**0.04**	41.93	**0.15**	7.85	**0.15**	5.99	**0.07**	6.22	**0.21**	1.18
*T*_*cw*_	44	64	0.00	0.07	**0.16**	39.13	**0.15**	7.76	**0.34**	5.27	**0.19**	5.76	**0.44**	0.96
herb (134 plots)														
*T*_*cw*_	8	53	0.01	0.12	0.02	65.00	**0.09**	9.65	0.01	7.51	0.02	6.81	**0.11**	1.68
*T*_*acw*_	8	53	0.00	0.12	**0.04**	64.33	0.01	10.13	0.00	7.55	0.00	6.85	**0.04**	1.76
*T*_*cw*_	34	53	**0.27**	0.10	**0.33**	53.45	**0.44**	7.69	**0.33**	6.12	**0.22**	6.00	**0.40**	1.34
woody (72 plots)														
*T*_*cw*_	5	11	**0.29**	0.02	**0.24**	35.68	**0.12**	8.34	**0.38**	3.55	**0.19**	3.44	**0.13**	0.55
*T*_*acw*_	5	11	**0.14**	0.02	**0.18**	37.08	**0.11**	8.36	**0.28**	3.82	**0.12**	3.58	**0.08**	0.57
*T*_*cw*_	10	11	**0.50**	0.01	**0.38**	33.06	**0.29**	7.48	**0.41**	3.47	**0.24**	3.35	**0.30**	0.49

Since RPP adjustment did not improve the linear fit between the mean trait values of vegetation and modern pollen, the reconstruction of temporal changes in leaf traits using Holocene pollen assemblages focused on leaf C and N content, leaf C/N ratio and leaf δ^13^C using pollen counts without RPP correction.

### Temporal changes in trait composition and comparison to modern conditions

**Fig 4:**
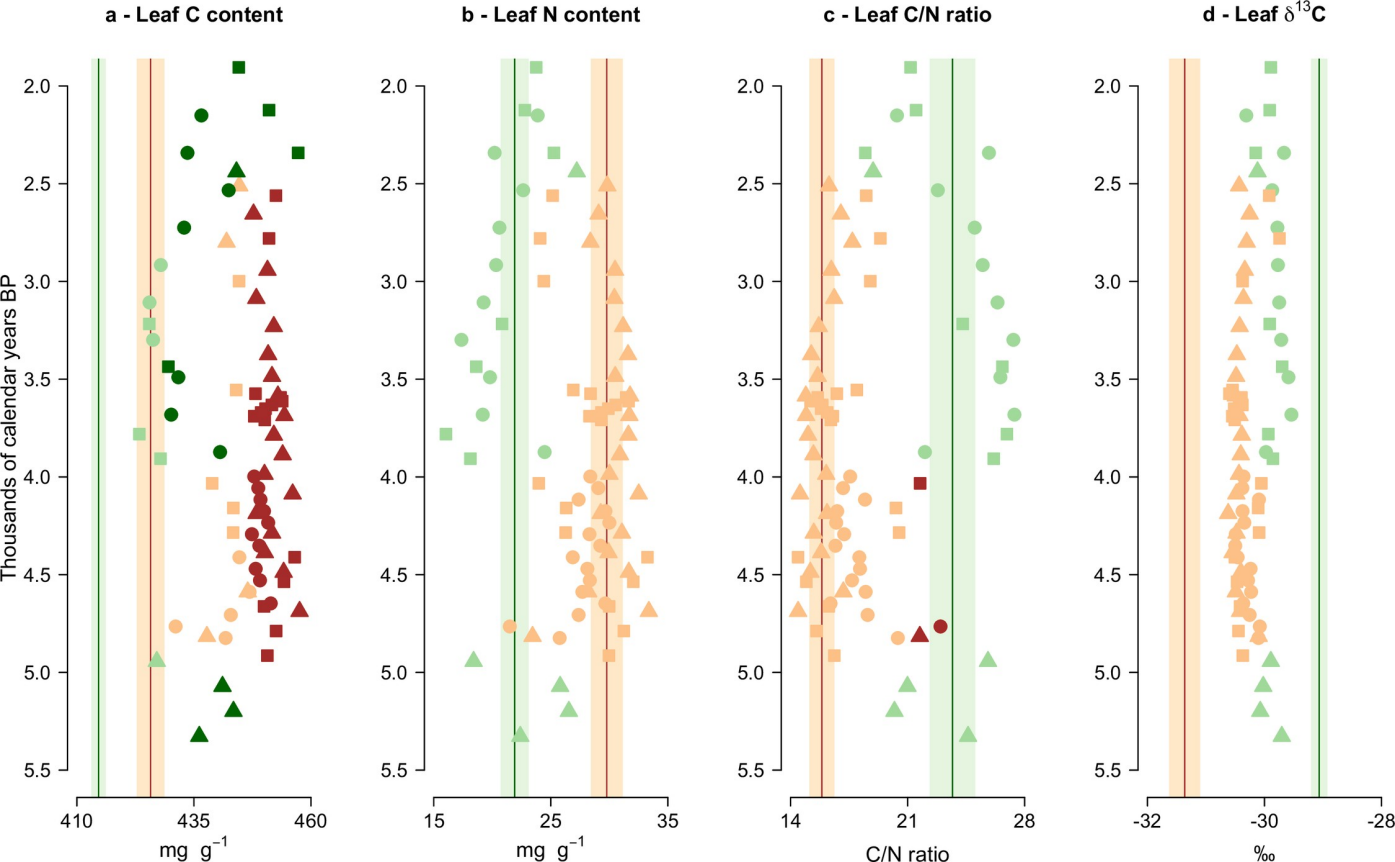
Changes in trait community weighted means (CWMs) through time (in thousands of calendar years before present) of the Holocene pollen-derived leaf trait records from three Romney Marsh sites (circles = Brookland; triangles = The Dowells; squares = Hope Farm). Green and brown symbols indicate the predominance of herbaceous and woody taxa in the Holocene record, respectively. The means of the modern vegetation (i.e., plant-based data) are shown for herb (green vertical lines) and woody (brown vertical lines) fens. Shaded areas around the modern means depict their 95% confidence intervals. Darker-coloured symbols show Holocene samples significantly different from their equivalent (herbaceous or woody) modern vegetation means at *p* < 0.05 (one-observation *t*-tests).

**Fig 5:**
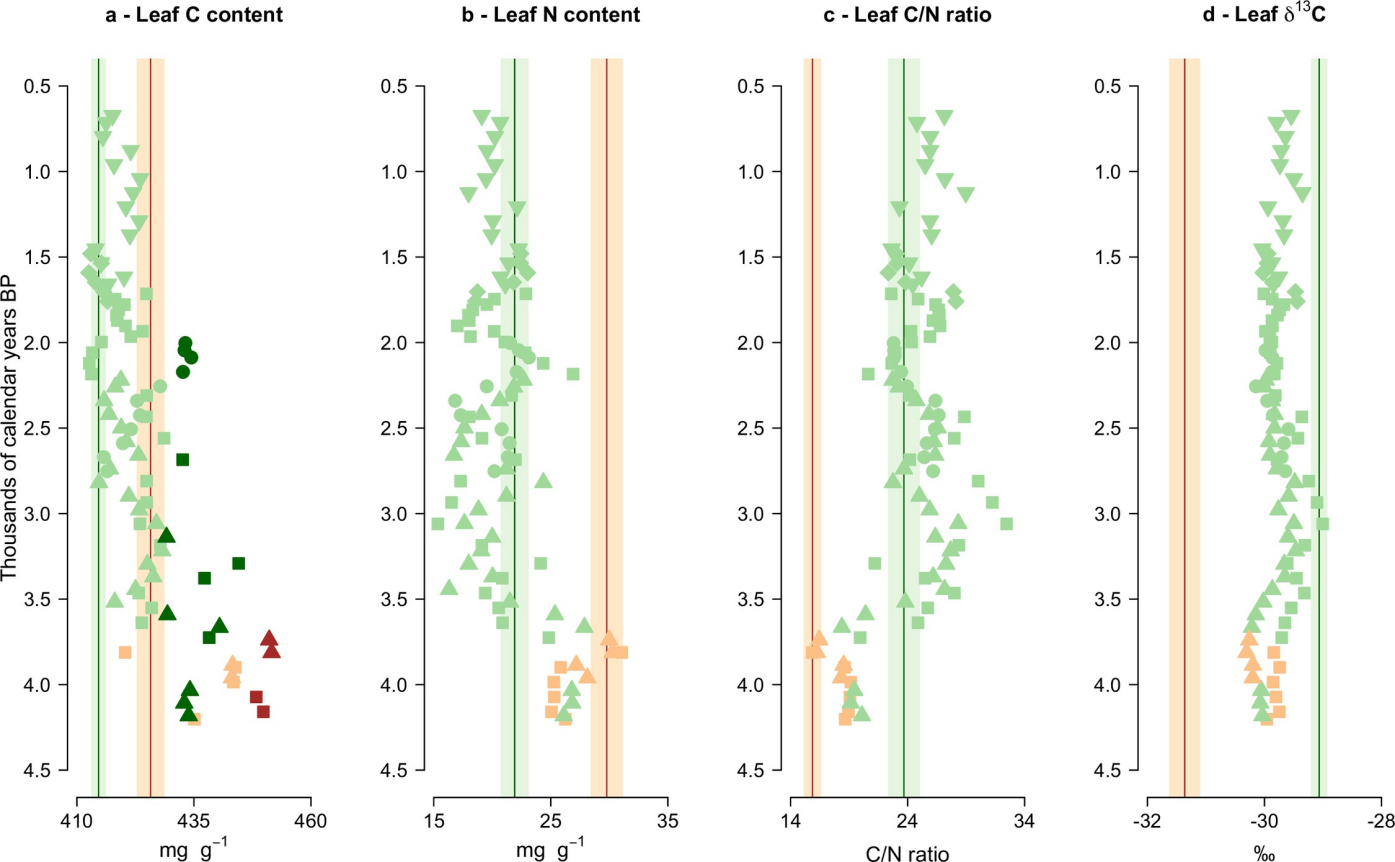
Changes in trait community weighted means (CWMs) through time (in thousands of calendar years before present) of the Holocene pollen-derived leaf trait records from five Fenland sites (circles = Murrow; squares = Redmere; diamonds = Swineshead; point-up triangles = Welney Washes 3^rd^ peat; point-down triangles = Welney Washes 4^th^ peat). Green and brown symbols indicate the predominance of herbaceous and woody taxa in the Holocene record, respectively. The means of the modern vegetation (i.e., plant-based data) are shown for herb (green vertical lines) and woody (brown vertical lines) fens. Shaded areas around the modern means depict their 95% confidence intervals. Darker-coloured symbols show Holocene samples significantly different from their equivalent (herbaceous or woody) modern vegetation means at *p* < 0.05 (one-observation *t*-tests).

Holocene trait reconstructions from Romney Marsh and Fenland are shown in Figs 4 and 5, respectively. Holocene pollen assemblages classed as woody (dominated by *Alnus* pollen, with variable amounts of *Quercus*, *Betula* and *Salix*) or herbaceous (with high Poaceae and Cyperaceae values) from Romney Marsh between *c*. 5300–2500 cal. yrs. BP displayed consistently different leaf C and N values (Fig 4A and 4B). The woody Holocene communities largely showed higher leaf C than the herbaceous Holocene assemblages (Fig 4A), along with higher leaf N (Fig 4B), but a lower leaf C/N ratio (Fig 4C). The herbaceous Holocene samples showed slightly higher leaf δ^13^C values than the Holocene wooded samples (Fig 4D), likely due to higher leaf δ^13^C in the sampled C_3_ grasses and sedges than in the sampled trees.

Leaf C content of most of the Romney Marsh Holocene communities was significantly higher (*p* < 0.05; one-observation *t*-test) than their respective (wooded and herbaceous) contemporary communities (Fig 4A). This was likely caused by the presence of species with relatively high leaf C in the Holocene record (e.g., trees, shrubs and monocotyledon species), compared to the dicotyledon forbs with low leaf C that were comparatively abundant in the modern vegetation. Leaf N (Fig 4B) and leaf C/N ratio (Fig 4C) of the Holocene samples generally presented similar values to their respective (wooded and herbaceous) contemporary communities between *c*. 5300 and 1900 cal. yrs. BP, though the wooded assemblages from *c*. 4800 cal. yrs. BP at The Dowells and in the Brookland core and from *c*. 4000 cal. yrs. BP in the Hope Farm record showed significantly higher leaf C/N ratio (*p* < 0.05; one-observation *t*-test) than contemporary fen woodlands (Fig 4C), suggesting higher dominance of taxa with relatively carbon-rich leaves and recalcitrant leaf litter during those periods than at present. The Holocene herb communities revealed consistently lower leaf δ^13^C than the contemporary herb fens, while the Holocene woody samples showed higher leaf δ^13^C than the modern wooded fens, though differences were not significant (*p* > 0.05; one-observation *t*-test; Fig 4D). Regional arboreal pollen not originating from local assemblages may have driven down leaf δ^13^C values of the Holocene herbaceous samples, given the sampled trees have lower leaf δ^13^C on average than the sampled grasses and sedges. On the other hand, the presence of herbaceous pollen (especially Poaceae and Cyperaceae) in the Holocene pollen assemblages classified as woody might have resulted in the leaf δ^13^C values of the wooded pollen samples being higher than the modern woody fens.

The Fenland sites (Fig 5) were characterised by a relatively brief period of woodland dominance between *c*. 4200 and 3700 cal. yrs. BP, with no obvious associated change in leaf elemental traits, in contrast to the Romney Marsh sites. The pollen signal for these sites implies a relatively open carr woodland with herbaceous understorey, unlike the dense canopy and minimal understorey today. A long period when Poaceae and Cyperaceae pollen and pteridophyte spores dominated the fossil record followed (from *c*. 3700 to 700 cal. yrs. BP; Fig 5). These herbaceous assemblages showed similar values of leaf δ^13^C to the earlier woody assemblages (Fig 5D), but generally lower leaf C (Fig 5A) and leaf N (Fig 5B) and higher leaf C/N ratio (Fig 5C).

Leaf N (Fig 5B), leaf C/N ratio (Fig 5C) and leaf δ^13^C (Fig 5D) of the Holocene assemblages from Fenland did not deviate significantly from their contemporary equivalent communities (*p* > 0.05; one-observation *t*-test). However, leaf C (Fig 5A) values estimated from the woody Holocene samples from *c*. 4200 to 3700 cal. yrs. BP were significantly higher (*p* < 0.05; one-observation *t*-test) than modern fen woodlands. The herbaceous Holocene assemblages also revealed higher leaf C than modern herb fens during multiple time intervals (Fig 5A).

## Discussion

Understanding temporal changes in leaf traits with links to ecosystem processes and the competitive ability of species allows for the interpretation of changes in ecosystem functioning and community structure over time. Pollen assemblages from peat samples have lower taxonomic diversity and a larger, less well defined spatial resolution than a vegetation survey centred on the same location. Despite these challenges, a clear community-level trait signal is present in the dataset for some leaf traits. Comparing community-level traits derived from vegetation survey species lists with those derived from pollen assemblages for six selected leaf traits showed that their linear fit was mainly dependent on the pollen count-weighting method used and whether herbaceous and woody taxa were analysed separately or in combination. Despite a statistically significant linear relationship in almost all cases, woody taxa consistently showed better linear fits between modern pollen and vegetation than herbaceous taxa and all taxa in combination. The relatively poor linear fits shown by herbaceous taxa may have been caused by trait averaging of six indistinguishable pollen (spore) types (Apiaceae, *Cirsium*-type, Cyperaceae, *Mentha*-type, Poaceae and Pteropsida), which reduced pollen-derived trait variability in relation to the vegetation-derived data. On the other hand, only one woody pollen type was averaged (*Salix*-type), resulting in a better fit with the vegetation data due to lower loss of trait variability. Variations over time in community traits derived from pollen assemblages can be confidently interpreted as showing variations in the original community traits of the vegetation around the sampled point. In our study, leaf nutrient traits proved the most promising in reconstructions from pollen assemblages preserved in sedimentary archives. Reconstructing leaf nutrient status from peatland records in wetlands may allow for inferences to be made about past peat forming conditions (e.g., soil C accumulation rates based on leaf nutrient composition of the dominant taxa).

### Linking trait reconstructions to ecosystem processes

The reconstruction of vegetation trait patterns from Holocene assemblages has shown that the differences between herbaceous and woody Holocene assemblages and between Holocene and modern fen communities may represent differences in nutrient acquisition strategies between different life forms. Resource-use strategy will have implications for ecosystem processes through changes in leaf litter quality, which has been found to differ consistently across plant functional groups in peatlands [59] and to be more important in determining biomass accumulation and decomposition rates than environmental factors [60]. Given their generally higher leaf C/N ratio, periods dominated by monocot herbaceous vegetation in the mid- to late-Holocene may have promoted enhanced soil C retention compared to wooded periods. However, some wooded assemblages in the mid-Holocene in Fenland and in the late-Holocene in Romney Marsh may also have experienced enhanced peat accumulation rates and soil C retention due to their significantly higher leaf C content than those seen for average contemporary fen communities. Higher leaf C is normally the characteristic of species with thick, N-poor leaves that are rich in recalcitrant substances and known to promote low leaf digestibility and decomposition rates [22]. Therefore, it is possible these communities experienced lower C loss from soils during such periods when compared to present-day conditions. On the other hand, the relatively high-quality litter input of the forbs found in relative abundance in the contemporary woody fens is easily decomposed and can increase rates of nitrification [61] and heterotrophic respiration [62], promoting nutrient uptake and C loss from soil. Indeed, such conditions seem to favour soil microbial communities dominated by bacteria that perform rapid rates of mineralisation and nitrification [63].

The current analyses only considered leaf traits of vascular plants, but given the importance of below ground organs of wetland species in adapting to waterlogged and nutrient poor conditions [64], quantifying temporal changes of belowground traits would be a promising development. Additional plant traits need to be investigated through modern trait studies and applying the methods used here to determine which above- and belowground traits are generally important for driving changes in ecosystem processes over long ecological timescales [9].

### Linking plant traits to pollen data

Issues related to linking plant traits to pollen data include the limited taxonomic resolution to which pollen can be identified, which hinder the ability to assign trait values to indistinguishable pollen types and to capture interspecific functional differences within and across communities. There are also difficulties concerning the pollen-plant abundance relationship and pollen source area and representation [65], which are pertinent for abundance-weighted measures to describe the functional structure of heterogeneous habitats. Results from the vegetation and modern pollen comparisons were somewhat equivocal. Our analyses may have been hindered by choice of traits. Future investigations should include traits based on plant organs other than leaves. We believe the comparison between modern pollen and modern vegetation is an important step that has been often omitted from previous similar studies. It is sensible to base reconstruction studies on modern data that will then allow for extrapolation to other sites. One step that may be included to validate the current methodology is the comparison between geochemical data collected from peat and pollen-derived trait data (e.g., C- and N-based nutrient data) collected from the same Holocene deposit to examine the extent to which Holocene pollen captures a local fen vegetation (in addition to regional) component. It can be argued that the pollen signal from fen communities has a very strong local component [40], especially in communities with a tree canopy. If the local signal is stronger than the long-distance signal within the pollen assemblages, the peat geochemical and pollen-derived data should show similar trends over time. However, trait data from all plant organs must be available for such comparison since peat sediments will include the remains of whole plants, not only leaves [66].

A current theme in pollen analysis research is the quantitative reconstruction of past land cover using models of the relationship between pollen assemblages and the surrounding vegetation to correct for the pollen production and spatial dispersal issues identified above (e.g., [5, 67, 68]). However, in our case the adjustment of pollen counts to account for pollen productivity did not improve the linear relationship between modern pollen and vegetation. One reason might be that the RPP values used here could be inappropriate for plants growing in fen conditions. Most RPP estimates used in deriving standard sets are based on studies of moss polsters or lake sediments from largely dry landscapes with mixed vegetation of farmland, grazing land, woodland and sometimes heathland. A palynologically equivalent (p.e.) taxon with a single pollen source plant species (e.g., *Alnus glutinosa*) may well produce different amounts of pollen when growing as the dominant taxon in a regularly inundated fen woodland rather than as a minor component of a mixed woodland at a drier location (e.g., [42]). In addition, many p.e. taxa represent a mixture of plant species (e.g., Cyperaceae). The taxonomic mixture in the areas studied to estimate the general values used may not be typical of those found in fen landscapes. In addition, different management regimes may affect flowering behaviour. For instance, Poaceae may have flowering suppressed by grazing or environmental stresses like shading within a woodland setting. A better understanding of the environmental controls on RPP values and collection of estimates using samples from peat-forming habitats to determine whether RPP values are indeed different would, we believe, provide a better basis for the type of trait reconstruction model calibration considered here.

The mismatch in spatial scale between the vegetation survey area and the pollen source area of the moss samples will also contribute to the errors seen in the modern linear models. The pollen source area of a moss sample can be defined in multiple ways, but in practical terms the source area of low-growing herbaceous vegetation is generally smaller than that of taller trees for a given sample. The presence or absence of tree canopy has a dramatic effect on pollen source area, since its absence allows for a larger component of long-distance pollen to be added to the forming assemblage. In our study, model fit was strongest when arboreal taxa were considered alone, which may be partly due to a smaller dataset–all samples included came from below-canopy peat-forming contexts, meaning a smaller overall pollen source area and greater similarity of pollen source areas across the dataset. In addition, samples from wooded sites have a higher proportion of tree pollen that mostly represent only one modern plant taxon, reducing the smoothing effect of the more varied palynological equivalent types. Adding in the samples from open contexts increases dataset size and range of trait values, but also the variety of pollen source area and the proportion of palynological equivalents taxa where trait values are derived from an average of values from multiple plant species. In order to develop a more effective reconstruction tool, a two-part process may be required: first, using the peat structure, non-pollen palynomorphs and macrofossils to identify the broad fen context and second, applying reconstruction algorithms calibrated to that particular environment.

The method presented here adds to the analytical techniques available for modelling ecosystem interactions over extended timescales, particularly complex direct and indirect habitat-trait relationships that determine plant community structure and composition and influence ecosystem processes. Our study demonstrates it is possible to reconstruct trends in functional trait composition from pollen assemblages preserved in sedimentary archives and offers a promising approach for studying how temporal changes in functional traits can potentially affect ecosystem functioning. Limitations commonly associated with conventional pollen analysis need to be addressed, particularly issues regarding the estimation of plant abundances from pollen count data. Although traits could be inferred from the sediment using geochemistry or macrofossil remains when available (e.g., peat may be too humified for macrofossil preservation), the advantage of using pollen analysis is the spatial scale at which the reconstruction of vegetation trait patterns can be made, as sediment-based reconstructions only represent *in-situ* conditions. Future development of this approach should consider the use of modeling algorithms to more accurately reconstruct vegetation abundances at regional spatial scales, to improve confidence in estimated values of abundance-weighted measures of trait diversity and composition. This seems to be a promising field of future research worthy of further investigation.

## Supporting information

S1 Fig Vegetation communities of Woodwalton Fen (A) and Upton Broad (B), with the location of the sites and the Romney Marsh area and the Fenland basin in England (C).
(DOCX)

S1 Table Description of the Holocene pollen sites in Romney Marsh and Fenland. (DOCX)

S2 Table Palynological equivalents. (DOCX)

S3 Table Trait means and standard deviations of multispecies modern pollen types. (DOCX)

S4 Table Trait means and standard deviations of multispecies Holocene pollen types. (DOCX)

S1 Appendix Vegetation survey methods. (DOCX)

S2 Appendix Description of the Holocene sites in Romney Marsh and Fenland. (DOCX)

S3 Appendix Plant trait measurements. (DOCX)

S1 Data File Plant leaf trait data. (XLSX)

S2 Data File Vegetation abundance data. (XLSX)
