# Supplementary material for: A method for reconstructing temporal changes in vegetation functional trait composition using Holocene pollen assemblages
Source: PLoS One. 2019 May 29;14(5):e0216698. doi: 10.1371/journal.pone.0216698 (PMC6541253; doi:10.1371/journal.pone.0216698)
Supplement: S1 Fig — Vegetation communities of Woodwalton Fen (A) and Upton Broad (B), with the location of the sites and the Romney Marsh area and the Fenland basin in England (C). (DOCX) [file pone.0216698.s001.docx]

**A method for reconstructing temporal changes in vegetation functional trait composition using Holocene pollen assemblages**

*PLOS ONE*

Fabio Carvalho, Kerry A. Brown, Martyn P. Waller, M. Jane Bunting, Arnoud Boom and Melanie J. Leng

Corresponding author: Fabio Carvalho ([fabiocgs@yahoo.com](mailto:fabiocgs@yahoo.com))


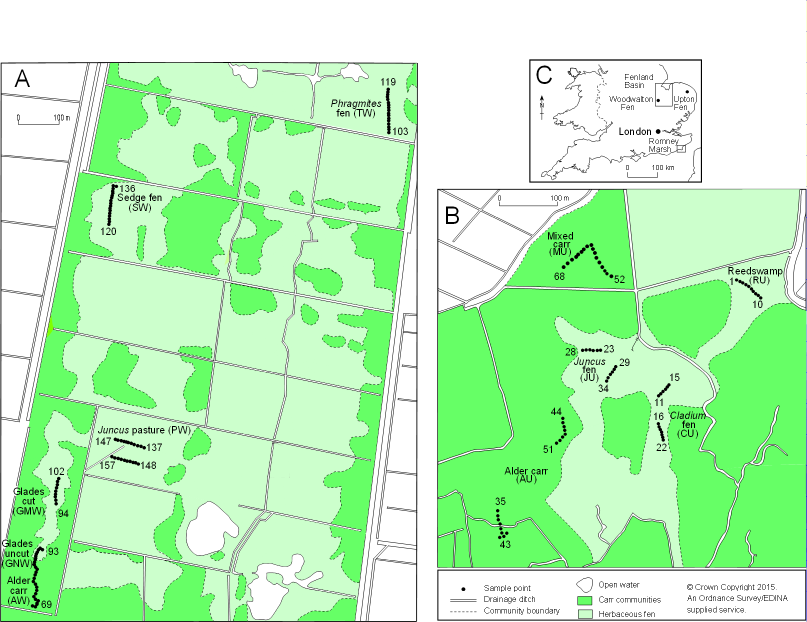


**S1 Fig: Vegetation communities of Woodwalton Fen (A) and Upton Broad (B), with the location of the sites and the Romney Marsh area and the Fenland basin in England (C).** Black circles are the sampling plots, with community names and plot numbers shown for each plant community. Sampling plots of the *Juncus* pasture (plots 137 to 157) were not used in these analyses.
