# Supplementary material for: A method for reconstructing temporal changes in vegetation functional trait composition using Holocene pollen assemblages
Source: PLoS One. 2019 May 29;14(5):e0216698. doi: 10.1371/journal.pone.0216698 (PMC6541253; doi:10.1371/journal.pone.0216698)
Supplement: S1 Table — (DOCX) [file pone.0216698.s002.docx]

**A method for reconstructing temporal changes in vegetation functional trait composition using Holocene pollen assemblages**

*PLOS ONE*

Fabio Carvalho, Kerry A. Brown, Martyn P. Waller, M. Jane Bunting, Arnoud Boom and Melanie J. Leng

Corresponding author: Fabio Carvalho ([fabiocgs@yahoo.com](mailto:fabiocgs@yahoo.com))

**S1 Table: Description of the Holocene pollen sites in Romney Marsh and Fenland.**

**S1 Table: Description of the Holocene pollen sites in Romney Marsh and Fenland.** The major taxa in the local pollen assemblage zones (LPAZs) reflect different types of vegetation at that particular locality. Derived from Waller [1] and Waller et al. [2].

**References**

1. Waller MP. The Fenland Project, Number 9: Flandrian environmental change in Fenland. Monograph No. 70. Cambridge, UK: East Anglian Archaeology; 1994. 353 p.

2. Waller MP, Long AJ, Long D, Innes JB. Patterns and processes in the development of coastal mire vegetation: multi-site investigations from Walland Marsh, Southeast England. Quaternary Sci Rev. 1999;18(12):1419-44.
