# Supplementary material for: A method for reconstructing temporal changes in vegetation functional trait composition using Holocene pollen assemblages
Source: PLoS One. 2019 May 29;14(5):e0216698. doi: 10.1371/journal.pone.0216698 (PMC6541253; doi:10.1371/journal.pone.0216698)
Supplement: S2 Table — (DOCX) [file pone.0216698.s003.docx]

**A method for reconstructing temporal changes in vegetation functional trait composition using Holocene pollen assemblages**

*PLOS ONE*

Fabio Carvalho, Kerry A. Brown, Martyn P. Waller, M. Jane Bunting, Arnoud Boom and Melanie J. Leng

Corresponding author: Fabio Carvalho ([fabiocgs@yahoo.com](mailto:fabiocgs@yahoo.com))

**S2 Table: Palynological equivalents table.** A hyphen indicates absence. If a taxon was recorded in the vegetation then the pollen type is the ‘palynological equivalent’ (p.e.). The taxonomic precision to which identification of the pollen was possible varied (with preservation) and, where indicated by brackets, some of the grains likely to have been derived from that species may also have been included in a p.e. with a lower level of taxonomic resolution. ^1^Taxa recorded at < 4 locations or with < 4 hits in the vegetation survey. U = Upton Broad and W= Woodwalton Fen. Species order follows trees, shrubs, climbers, herbs and pteridophytes.

| **Species recorded in the vegetation** | **Site** | **Pollen type** | **Site** |
| --- | --- | --- | --- |
| - | - | *Abies* | U |
| - | - | *Picea* | U, W |
| - | - | *Pinus* | U, W |
| - | - | *Ulmus* | U, W |
| - | - | *Fagus sylvatica* | U, W |
| - | - | *Castanea sativa* | U |
| *Quercus robur* | U, W | *Quercus* | U, W |
| *Betula pubescens* | U, W | *Betula* | U, W |
| *Alnus glutinosa* | U, W | *Alnus glutinosa* | U, W |
| *-* |  | *Carpinus betulus* | U, W |
| *-* |  | *Tilia* | U, W |
| *-* |  | *Taxus baccata* | U, W |
| *Ilex aquifolium* | U | *Ilex aquifolium* | U |
| *Acer pseudoplatanus* | U | *Acer* | U, W |
| *Fraxinus excelsior* | U | *Fraxinus excelsior* | U, W |
| *-* |  | *Corylus avellana* | U, W |
| *S2 Table continued* |  |  |  |
| **Species recorded in the vegetation** | **Site** | **Pollen type** | **Site** |
| *Salix caprea* | U | *Salix* | U, W |
| *Salix cinerea* | U, W | *Salix* | U, W |
| *Salix fragilis*^1^ | U | *Salix* | U, W |
| *Salix repens* | U | *Salix* | U, W |
| *Crataegus monogyna* | U, W | *Sorbus*-type | U, W |
| *Prunus padus* | U | *Sorbus*-type | U, W |
| *Sorbus aucuparia* | U | *Sorbus*-type | U, W |
| *-* |  | *Cornus sanguinea* | U |
| *Frangula alnus*^1^ | U | *Frangula alnus* | U, W |
| *Rhamnus cathartica*^1^ | W | *Rhamnus cathartica* | U, W |
| *-* |  | *Ligustrum vulgare* | U, W |
| *-* |  | *Sambucus nigra* | U, W |
| *Viburnum opulus* | U | *Viburnum opulus* | U, W |
| *Myica gale* | U | *Myrica gale* | U, W |
| *Lonicera periclymenum* | U | *Lonicera periclymenum* | U, W |
| *Hedera helix* | U | *Hedera helix* | U, W |
| *Calystegia sepium* | U, W | *Calystegia* | U, W |
| *-* |  | *Convolvulus* | W |
| *Humulus lupulus* | U | *Humulus lupulus* | U, W |
| *-* |  | *Bryonia dioica* | U |
| *Tamus communis*^1^ | U | - |  |
| *-* |  | *Caltha palustris*-type | U, W |
| *Ranunculus acris* | W | *Ranunculus acris*-type | U, W |
| *Ranunculus ficaria*^1^ | U | *Ranunculus acris*-type | U, W |
| *Ranunculus flammula* | W | *Ranunculus acris*-type | U, W |
| *Ranunculus repens* | U, W | *Ranunculus acris*-type | U, W |
| *Thalictrum flavum* | W | *Thalictrum* | U |
| *Ceratocapnos claviculata*^1^ | U | *-* |  |
| *Urtica dioica* | U, W | *Urtica* | U, W |
| - |  | Chenopodiaceae | U, W |
| *Cerastium fontanum*^1^ | W | Caryophyllaceae undiff. | U, W |
| *Moehringia trinervia* | U, W | Caryophyllaceae undiff. | U, W |
| *Stellaria media* | W | Caryophyllaceae undiff. | U, W |
| *Stellaria palustris* | W | Caryophyllaceae undiff. | U, W |
| *Stellaria uliginosa*^1^ | U | Caryophyllaceae undiff. | U, W |
| *Lychnis flos-cuculi* | W | Caryophyllaceae undiff. | U, W |
| *Silene dioica*^1^ | U | Caryophyllaceae undiff. | U, W |
| *-* |  | *Persicaria maculosa*-type | U, W |
| *-* |  | *Polygonum* | U, W |
| *S2 Table continued* |  |  |  |
| **Species recorded in the vegetation** | **Site** | **Pollen type** | **Site** |
| *-* |  | *Rumex acetosa* | U, W |
| *Rumex crispus*^1^ | W | *Rumex obtusifolius*-type | U, W |
| *Rumex sanguineus* | W | *Rumex sanguineus*-type | U, W |
| *Hypericum tetrapterum* | U | *Hypericum perforatum*-type | U, W |
| *Viola sp.*^1^ | W | *Viola palustris*-type | U, W |
| *Cardamine flexuosa* | U, W | Brassicaceae | U, W |
| *Cardamine pratensis* | W | Brassicaceae | U, W |
| *Ribes nigrum* | U | *Ribes* | U, W |
| *Ribes rubrum* | U, W | *Ribes* | U, W |
| *-* |  | *Calluna vulgaris* | U, W |
| *Lysimachia vulgaris* | U, W | *Lysimachia vulgaris*-type | U, W |
| *-* |  | *Chrysosplenium* | U |
| *Filipendula ulmaria* | U, W | *Filipendula* | U, W |
| *Rubus fruticosus* agg*.* | U, W | *Rubus* undiff. (Rosaceae undiff.) | U, W |
| *Potentilla anserina* | W | *Potentilla*-type | U, W |
| *Potentilla erecta* | W | *Potentilla*-type | U, W |
| *Potentilla palustris*^1^ | U | *Potentilla*-type | U, W |
| *Geum urbanum*^1^ | U | - |  |
| *Rosa* sp. | U, W | *Rosa* (Rosaceae undiff.) | U, W |
| *Lotus pedunculatus* | U, W | *Lotus* | U, W |
| *Vicia cracca* | W | *Vicia cracca* (Fabaceae undiff.) | W |
| *-* |  | *Vicia sylvatica*-type (Fabaceae undiff.) | U, W |
| *-* |  | *Lathyrus* (Fabaceae undiff.) | U, W |
| *Trifolium repens*^1^ | W | *Trifolium*-type (Fabaceae undiff.) | W |
| *Lythrum salicaria* | U, W | *Lythrum salicaria* | U, W |
| *Circaea lutetiana* | U, W | *Circaea* | W |
| *Epilobium hirsutum* | U, W | *-* |  |
| *Epilobium montanum* | W | *-* |  |
| *Epilobium palustre* | U, W | *-* |  |
| *-* |  | *Mercurialis perennis* | W |
| *-* |  | *Euphorbia* | U |
| *Geranium robertianum* | U, W | *Geranium* | U, W |
| *Impatiens* sp.^1^ | W | *-* |  |
| *Hydrocotyle vulgaris* | W | *Hydrocotyle vulgaris* | U, W |
| *Angelica sylvestris* | U, W | Apiaceae undiff. | U, W |
| *Apium nodiflorum*^1^ | U, W | Apiaceae undiff. | U, W |
| *Sium latifolium* | U | Apiaceae undiff. | U, W |
| *Peucedanum palustre* | U | Apiaceae undiff. | U, W |
| *Solanum dulcamara* | U, W | *Solanum dulcamara* | U, W |
| *S2 Table continued* |  |  |  |
| **Species recorded in the vegetation** | **Site** | **Pollen type** | **Site** |
| *-* |  | *Echium vulgare* | U |
| *Symphytum officinale* | W | *Symphytum* | U, W |
| *Myosotis scorpioides* | W | *Myosotis arvensis*-type | U, W |
| *Stachys palustris* | W | Lamiaceae undiff. | U, W |
| *Stachys sylvatica* | W | Lamiaceae undiff. | U, W |
| *Lamium purpurea*^1^ | W | Lamiaceae undiff. | U, W |
| *Scutellaria galericulata* | U, W | - |  |
| *Glechoma hederacea* | U, W | - |  |
| *Prunella vulgaris* | W | - |  |
| *Lycopus europaeus* | W | *Mentha*-type | U, W |
| *Mentha aquatica* | U, W | *Mentha*-type | U, W |
| *-* |  | *Plantago major* | U, W |
| *-* |  | *Plantago lanceolata* | U, W |
| *-* |  | *Scrophularia-type* | U, W |
| *Veronica anagallis-aquatica*^1^ | U | *Veronica* | U, W |
| *Veronica scutellata*^1^ | W | *Veronica* | U, W |
| *Odontites vernus*^1^ | W | - |  |
| *Galium aparine* | U, W | Rubiaceae | U, W |
| *Galium palustre* | W | Rubiaceae | U, W |
| *Galium uliginosum* | U, W | Rubiaceae | U, W |
| *Valeriana officinalis* | U | *Valeriana officinalis* | U, W |
| *Cirsium arvense* | W | *Cirsium*-type | U, W |
| *Cirsium palustre* | U, W | *Cirsium*-type | U, W |
| *Cirsium vulgare*^1^ | W | *Cirsium*-type | U, W |
| *Centaurea nigra*^1^ | W | *Centaurea nigra* | U, W |
| *Lapsana communis*^1^ | W | Lactuceae | U, W |
| *Taraxacum* sp.^1^ | W | Lactuceae | U, W |
| *Sonchus* sp.^1^ | W | Lactuceae | U, W |
| *Eupatorium cannabinum* | U, W | Solidago virgaurea-type | U, W |
| *Senecio* sp.^1^ | W | Solidago virgaurea-type | U, W |
| *-* |  | *Artemisia*-type | U, W |
| *Achillea millefolium* | W | *Achillea*-type | U, W |
| *Juncus articulatus* | W | - |  |
| *Juncus bufonius*^1^ | W | - |  |
| *Juncus conglomeratus* | W | - |  |
| *Juncus effusus* | W | - |  |
| *Juncus inflexus*^1^ | W | - |  |
| *Juncus subnodulosus* | U, W | - |  |
| *Luzula multiflora* | W | - |  |
| *S2 Table continued* |  |  |  |
| **Species recorded in the vegetation** | **Site** | **Pollen type** | **Site** |
| *Cladium mariscus* | U, W | *Cladium mariscus* (Cyperaceae und.) | U, W |
| *Carex acutiformis* | U, W | Cyperaceae undiff. | U, W |
| *Carex diandra*^1^ | U | Cyperaceae undiff. | U, W |
| *Carex elata* | W | Cyperaceae undiff. | U, W |
| *Carex nigra*^1^ | W | Cyperaceae undiff. | U, W |
| *Carex otrubae* | W | Cyperaceae undiff. | U, W |
| *Carex panicea* | U, W | Cyperaceae undiff. | U, W |
| *Carex riparia* | U | Cyperaceae undiff. | U, W |
| *Carex rostrata* | W | Cyperaceae undiff. | U, W |
| *Carex viridula* | W | Cyperaceae undiff. | U, W |
| *Eleocharis palustris* | W | Cyperaceae undiff. | U, W |
| *Agrostis capillaris* | W | Poaceae undiff. | U, W |
| *Agrostis stolonifera* | U, W | Poaceae undiff. | U, W |
| *Anthoxanthum odoratum* | W | Poaceae undiff. | U, W |
| *Brachypodium sylvaticum*^1^ | W | Poaceae undiff. | U, W |
| *Calamagrostis canescens* | U, W | Poaceae undiff. | U, W |
| *Calamagrostis epigejos* | W | Poaceae undiff. | U, W |
| *Elytrigia repens* | W | Poaceae undiff. | U, W |
| *Festuca rubra* | W | Poaceae undiff. | U, W |
| *Holcus lanatus* | W | Poaceae undiff. | U, W |
| *Holcus mollis* | U | Poaceae undiff. | U, W |
| *Molinia caerulea* | W | Poaceae undiff. | U, W |
| *Poa trivialis* | U, W | Poaceae undiff. | U, W |
| *Poa pratensis*^1^ | W | Poaceae undiff. | U, W |
| *Phalaris arundinacea* | W | Poaceae undiff. | U, W |
| *Phragmites australis* | U, W | Poaceae undiff. | U, W |
| *Glyceria fluitans* | W | *Glyceria/Bromus* | U, W |
| *-* |  | Cerealia-type | U, W |
| *Dactylorhiza* sp.^1^ | W | - |  |
| *-* |  | *Myriophyllum spicatum* | U |
| *-* |  | *Callitriche* | U |
| *-* |  | *Alisma*-type | W |
| *-* |  | *Potamogeton natans*-type | W |
| *-* |  | *Sparganium emersum*-type | U, W |
| *Typha latifolia*^1^ | U | *Typha latifolia* | U, W |
| *Iris pseudacorus* | U, W | *Iris* | U, W |
| *Equisetum palustre* | U | *Equisetum* | U |
| *-* |  | *Osmunda regalis* | U, W |
| *S2 Table continued* |  |  |  |
| **Species recorded in the vegetation** | **Site** | **Pollen type** | **Site** |
| *-* |  | *Polypodium* | W |
| *-* |  | *Pteridium aquilinum* | W |
| *Thelypteris palustris* | U | *Thelypteris palustris* (Pteropsida (monolete) indet.) | U, W |
| *Athyrium filix-femina*^1^ | U | *Athyrium filix-femina* (Pteropsida (monolete) indet). | U |
| *Dryopteris filix-mas*^1^ | U | *Dryopteris filix-mas* (Pteropsida (monolete) indet). | U, W |
| *Dryopteris dilatata* | U, W | *Dryopteris dilatata* (Pteropsida (monolete) indet.) | U, W |
