# Supplementary material for: A method for reconstructing temporal changes in vegetation functional trait composition using Holocene pollen assemblages
Source: PLoS One. 2019 May 29;14(5):e0216698. doi: 10.1371/journal.pone.0216698 (PMC6541253; doi:10.1371/journal.pone.0216698)
Supplement: S3 Table — (DOCX) [file pone.0216698.s004.docx]

**A method for reconstructing temporal changes in vegetation functional trait composition using Holocene pollen assemblages**

*PLOS ONE*

Fabio Carvalho, Kerry A. Brown, Martyn P. Waller, M. Jane Bunting, Arnoud Boom and Melanie J. Leng

Corresponding author: Fabio Carvalho ([fabiocgs@yahoo.com](mailto:fabiocgs@yahoo.com))

**S3 Table: Trait means and standard deviations of multispecies modern pollen types.** Five modern pollen types in Upton and four in Woodwalton had several species with trait data available and were thus averaged for the calculation of mean trait values (see Methods section in the main text). The vegetation species comprising each pollen type in each site are listed below. SLA = specific leaf area; LDMC = leaf dry-matter content.

|  | **SLA (cm^2^ mg^-1^)** | **LDMC (mg g^-1^)** | **Leaf C (mg g^-1^)** | **Leaf N (mg g^-1^)** | **Leaf C/N ratio** | **Leaf δ^13^C (‰)** |
| --- | --- | --- | --- | --- | --- | --- |
| **Upton** |  |  |  |  |  |  |
| Apiaceae | 0.24 ± 0.08 | 213.1 ± 14.39 | 399.92 ± 3.38 | 25.74 ± 4.07 | 15.95 ± 2.33 | -30.87 ± 1.6 |
| Cyperaceae | 0.16 ± 0.03 | 306.79 ± 20.76 | 418.19 ± 14.57 | 16.51 ± 5.07 | 27.13 ± 9 | -28.95 ± 2.35 |
| Poaceae | 0.26 ± 0.22 | 390.21 ± 127.18 | 407.18 ± 10.34 | 27.06 ± 12.34 | 17.89 ± 8.23 | -30.42 ± 3.38 |
| Pteropsida | 0.25 ± 0.05 | 291.08 ± 16.98 | 422.24 ± 3.13 | 15.51 ± 0.8 | 27.25 ± 1.2 | -29.93 ± 0.43 |
| *Salix* | 0.12 ± 0.01 | 423.75 ± 51.64 | 456.84 ± 4.31 | 23.75 ± 2.14 | 19.35 ± 1.8 | -29.34 ± 0.44 |
| **Woodwalton** |  |  |  |  |  |  |
| *Cirsium* type | 0.21 ± 0.08 | 123.04 ± 44.62 | 337.7 ± 3.45 | 15.03 ± 1.07 | 22.53 ± 1.84 | -31.16 ± 0.47 |
| Cyperaceae | 0.18 ± 0.06 | 342.56 ± 45.3 | 418 ± 14.07 | 14.77 ± 5.58 | 31.84 ± 12.59 | -29.63 ± 1.5 |
| *Mentha* type | 0.28 ± 0.02 | 209.02 ± 15.68 | 420.45 ± 19.23 | 21.02 ± 6.3 | 20.8 ± 5.32 | -32.25 ± 1.23 |
| Poaceae | 0.23 ± 0.16 | 380.69 ± 105.06 | 409.29 ± 15.13 | 22.75 ± 9.66 | 22.52 ± 8.13 | -30.02 ± 2.55 |

**Species composition of multispecies modern pollen types in Upton:**

Apiaceae: *Angelica sylvestris* and *Peucedanum palustre*

Cyperaceae: *Carex acutiformis*, *Carex panicea* and *Carex riparia*

Poaceae: *Agrostis stolonifera*, *Calamagrostis canescens*, *Phragmites australis* and *Poa trivialis*

Pteropsida: *Dryopteris dilatata* and *Thelypteris palustris*

*Salix*: *Salix caprea*, *Salix cinerea* and *Salix repens*

**Species composition of multispecies modern pollen types in Woodwalton:**

*Cirsium* type: *Cirsium arvense* and *Cirsium palustre*

Cyperaceae: *Carex acutiformis*, *Carex elata*, *Carex panicea* and *Carex viridula*

*Mentha* type: *Lycopus eropaeus* and *Mentha aquatica*

Poaceae: *Agrostis stolonifera*, *Calamagrostis canescens*, *Calamagrostis epigejos*, *Holcus lanatus*, *Molinia caerulea*, *Phalaris arundinacea*, *Phragmites australis* and *Poa trivialis*
