# Supplementary material for: A method for reconstructing temporal changes in vegetation functional trait composition using Holocene pollen assemblages
Source: PLoS One. 2019 May 29;14(5):e0216698. doi: 10.1371/journal.pone.0216698 (PMC6541253; doi:10.1371/journal.pone.0216698)
Supplement: S4 Table — (DOCX) [file pone.0216698.s005.docx]

**A method for reconstructing temporal changes in vegetation functional trait composition using Holocene pollen assemblages**

*PLOS ONE*

Fabio Carvalho, Kerry A. Brown, Martyn P. Waller, M. Jane Bunting, Arnoud Boom and Melanie J. Leng

Corresponding author: Fabio Carvalho ([fabiocgs@yahoo.com](mailto:fabiocgs@yahoo.com))

**S4 Table: Trait means and standard deviations of multispecies Holocene pollen types.** Seven multispecies pollen types in the Holocene record had several species with trait data available. The traits of the constituent species in each pollen type were averaged for the calculation of mean trait values per pollen type (see Methods section in the main text).

|  | **Leaf C (mg g^-1^)** | **Leaf N (mg g^-1^)** | **Leaf C/N ratio** | **Leaf δ^13^C (‰)** |
| --- | --- | --- | --- | --- |
| Apiaceae | 394.33 ± 9.97 | 26.28 ± 3.03 | 15.3 ± 2 | -31.13 ± 1.21 |
| *Cirsium* type | 337.7 ± 3.45 | 15.03 ± 1.07 | 22.53 ± 1.84 | -31.16 ± 0.47 |
| Cyperaceae | 422.25 ± 13.52 | 13.8 ± 5.18 | 34.77 ± 13.92 | -28.75 ± 1.79 |
| *Mentha* type | 420.45 ± 19.23 | 21.02 ± 6.3 | 20.8 ± 5.32 | -32.25 ± 1.23 |
| Poaceae | 409.29 ± 15.13 | 22.75 ± 9.66 | 22.52 ± 8.13 | -30.02 ± 2.55 |
| Pteropsida | 422.24 ± 3.13 | 15.51 ± 0.8 | 27.25 ± 1.2 | -29.93 ± 0.43 |
| *Salix* | 456.84 ± 4.31 | 23.75 ± 2.14 | 19.35 ± 1.8 | -29.34 ± 0.44 |

**Species composition of multispecies Holocene pollen types:**

Apiaceae: *Angelica sylvestris*, *Peucedanum palustre* and *Sium latifolium*

*Cirsium* type: *Cirsium arvense* and *Cirsium palustre*

Cyperaceae: *Carex acutiformis*, *Carex elata*, *Carex panicea*, *Carex riparia*, *Carex viridula* and *Cladium mariscus*

*Mentha* type: *Lycopus eropaeus* and *Mentha aquatica*

Poaceae: *Agrostis stolonifera*, *Calamagrostis canescens*, *Calamagrostis epigejos*, *Holcus lanatus*, *Molinia caerulea*, *Phalaris arundinacea*, *Phragmites australis* and *Poa trivialis*

Pteropsida: *Dryopteris dilatata* and *Thelypteris palustris*

*Salix*: *Salix caprea*, *Salix cinerea* and *Salix repens*
