# Supplementary material for: A method for reconstructing temporal changes in vegetation functional trait composition using Holocene pollen assemblages
Source: PLoS One. 2019 May 29;14(5):e0216698. doi: 10.1371/journal.pone.0216698 (PMC6541253; doi:10.1371/journal.pone.0216698)
Supplement: S1 Appendix — (DOCX) [file pone.0216698.s006.docx]

**A method for reconstructing temporal changes in vegetation functional trait composition using Holocene pollen assemblages**

*PLOS ONE*

Fabio Carvalho, Kerry A. Brown, Martyn P. Waller, M. Jane Bunting, Arnoud Boom and Melanie J. Leng

Corresponding author: Fabio Carvalho ([fabiocgs@yahoo.com](mailto:fabiocgs@yahoo.com))

**S1 Appendix: Vegetation survey methods**

Vegetation surveys were undertaken during the summers of 2013 and 2014. In order to record the spring ephemerals and perennials present, surveys were conducted in late May in the woodlands and between July and September in the open herbaceous communities. A stratified design (stratified by plant communities) was used to ensure a broad spatial representation of both fen carr and herbaceous fens (S1 Fig). Vascular plants were recorded using a point quadrat method [1] within 136 circular sampling plots of 2-m radius, spaced 6 metres apart along transect lines (S1 Fig). Plant cover was determined on a first ‘hit’ basis for each species (i.e., if more than one species was touched by the pin, each species was recorded as one ‘hit’).

The transect lines were located in a manner to ease relocation and mostly followed a south-north direction (S1 Fig). The survey consisted of dropping a plumb-bob into the centre of five rings of equal area at distances of 0.45, 1.08, 1.41, 1.67 and 1.89 m from the centre of the plot. These distances ensured equal weight to any point within the survey plot. Species not ‘hit’ during the field surveys, but present within the 2-m circle area, were also recorded. A total of 130 vascular plant species were present across the two fens, of which 103 were ‘hit’ during the surveys. The species ‘hit’ were used for abundance estimations for the calculations of plot-level weighted-mean trait values by scaling the number of ‘hits’ for a given species by the total number of ‘hits’ within the sampling plot.

**References**

1. Kent M, Paddy C. Vegetation description and analysis: a practical approach. London, UK: Belhaven Press; 1992.
