# Supplementary material for: A method for reconstructing temporal changes in vegetation functional trait composition using Holocene pollen assemblages
Source: PLoS One. 2019 May 29;14(5):e0216698. doi: 10.1371/journal.pone.0216698 (PMC6541253; doi:10.1371/journal.pone.0216698)
Supplement: S2 Appendix — (DOCX) [file pone.0216698.s007.docx]

**A method for reconstructing temporal changes in vegetation functional trait composition using Holocene pollen assemblages**

*PLOS ONE*

Fabio Carvalho, Kerry A. Brown, Martyn P. Waller, M. Jane Bunting, Arnoud Boom and Melanie J. Leng

Corresponding author: Fabio Carvalho ([fabiocgs@yahoo.com](mailto:fabiocgs@yahoo.com))

**S2 Appendix: Description of the Holocene sites in Romney Marsh and Fenland**

Pollen samples were extracted from mid- to late-Holocene peat deposits from eastern [1] and southern [2] England (Fenland and Romney Marsh, respectively) and are inferred from pollen and macro-remains (e.g., wood) to originate from herbaceous and woody vegetation (S1 Table). These Holocene assemblages derived from large sedimentary systems deposited during periods of widespread peat formation, with most of the peats intercalated between marine-brackish sediments. The selected locations were more than 1-km from contemporary dry land, so that the Holocene pollen assemblages were likely to be dominated by wetland taxa.

The three Romney Marsh sites (Brookland, The Dowells and Hope Farm; S1 Table) originate from the same peat layer that extended out from the upland edge from *c*. 5000 cal. yrs. BP onwards. They revealed initial sequences indicative of short periods of open vegetation, though this phase was more extended at The Dowells, the site closest to dry land. An extended period of tree pollen abundance followed at all three sites. The pollen assemblages in the upper half of the peat are more variable, though they are largely dominated by herbaceous pollen.

Four Fenland sites were selected (Murrow, Redmere, Welney Washes and Swineshead; S1 Table), including two peat profiles (the 3^rd^ and 4^th^ peats in stratigraphic sequence from the base upwards) at Welney Washes. At Welney Washes 3^rd^ peat and at Redmere, where the peat is overlain by freshwater marls rather than marine/brackish clays like the other sites, high pollen values of woody taxa were followed by assemblages dominated by herbaceous pollen. The opposite is true for Murrow. The Swineshead and the Welney Washes 4^th^ peat profiles were taken from the most recent stage of peat formation in Fenland and were probably deposited close to major rivers (the Witham and the Great Ouse). At both sites, the Holocene pollen assemblages were dominated by herbaceous taxa, with the presence within the peat of horizontally bedded *Phragmites* a strong indication of *in-situ* reedswamp at Swineshead. More detailed description of the sites are given in Waller [1], Waller et al. [2] and Waller et al. [3].

**References**

1. Waller MP. The Fenland Project, Number 9: Flandrian environmental change in Fenland. Monograph No. 70. Cambridge, UK: East Anglian Archaeology; 1994. 353 p.

2. Waller MP, Long AJ, Long D, Innes JB. Patterns and processes in the development of coastal mire vegetation: multi-site investigations from Walland Marsh, Southeast England. Quaternary Sci Rev. 1999;18(12):1419-44.

3. Waller M, Carvalho F, Grant MJ, Bunting MJ, Brown K. Disentangling the pollen signal from fen systems: modern and Holocene studies from southern and eastern England. Review of Palaeobotany and Palynology. 2017;238:15-33.
