# Supplementary material for: A method for reconstructing temporal changes in vegetation functional trait composition using Holocene pollen assemblages
Source: PLoS One. 2019 May 29;14(5):e0216698. doi: 10.1371/journal.pone.0216698 (PMC6541253; doi:10.1371/journal.pone.0216698)
Supplement: S3 Appendix — (DOCX) [file pone.0216698.s008.docx]

**A method for reconstructing temporal changes in vegetation functional trait composition using Holocene pollen assemblages**

*PLOS ONE*

Fabio Carvalho, Kerry A. Brown, Martyn P. Waller, M. Jane Bunting, Arnoud Boom and Melanie J. Leng

Corresponding author: Fabio Carvalho ([fabiocgs@yahoo.com](mailto:fabiocgs@yahoo.com))

**S3 Appendix: Plant trait measurements**

Leaf samples from vascular plants (varying from 1 to 7 per individual) were collected from sun-exposed healthy-looking adult individuals at Upton Broad (early September 2013) and Woodwalton Fen (early September 2014). The number of specimens collected per species varied between 1 and 19. A minimum of five specimens were collected for species with abundance category of 4 or higher in the Domin scale (following Rodwell [1]) at the community level, except for *Carex riparia* in the reedswamp in Upton (four specimens measured). A total of 69 species and just over 1300 individuals were sampled. Three of the species (*Ceratocapnos claviculata*, *Galium uliginosum* and *Rhamnus cathartica*) did not have sufficient plant material for elemental and isotopic measurements (see below), and were therefore not included in any trait analysis. Complete trait data were thus available for 66 species, of which *Frangula alnus* and *Sium latifolium*, though with fully measured traits, were not ‘hit’ during the surveys (i.e., no abundance cover calculation was possible). Therefore, a total of 64 measured species with abundance cover estimation were used in vegetation analyses involving trait data, representing 62.1% of the total number of species encountered across Upton and Woodwalton (103 species). This represented approximately 98% of the total species cover across the sampled sites (estimated by scaling the number of ‘hits’ with trait measurements by the total number of ‘hits’ overall). Upton had 97.4% of total species cover with trait measurements and Woodwalton 98.7%. The mean coverage per plot was 98%, and the minimum was 70.9% (one plot in the alder carr in Upton with relatively high abundance of *Prunus padus*, a species with no trait measurements). Seventy-five plots (55.1% of 136 plots) presented 100% trait coverage (i.e., all species encountered were sampled for trait measurements). Pakeman and Quested [2] suggested that an adequate weighted estimate of community-level quantitative single traits can be achieved by sampling the species comprising over 80% of the biomass, though sampling more species could be worthwhile in terms of accuracy. Only one plot had species with trait measurements representing less than 80% of total cover, while six plots (4.4% of 136 plots) had species with trait measurements representing less than 90% of total cover. Five of these were in the alder carr in Upton, where *Prunus padus*, *Holcus mollis* and *Sorbus aucuparia* showed relatively high abundances and had no trait measurements, while one plot in the sedge fen in Woodwalton revealed relatively high abundance of the grass *Anthoxanthum odoratum*, though no trait data were collected for that species. The high species cover with trait measurements presented here is most likely because all dominant tree species in the woodlands and all the main monocot species in the herbaceous sites (the most vigorous life forms in their respective communities) were sampled for trait measurements, confidently accounting for more than 80% of the biomass present in each community.

Plant leaf traits were determined following recently published standardised protocols [3]. Leaf samples were kept in moistened bags at under 4°C for up to 72 h prior to fresh leaf mass and leaf area measurements. Leaf lamina and petiole were used to determine fresh leaf area with a leaf area meter (LI-COR LI3000C), while leaf dry mass was computed after oven drying for 72 hours at 70°C. Oven-dried samples (leaf lamina only) were powdered by grinding and weighed to 5 ± 0.2 g. These samples were then analysed for leaf C and N concentrations (%C and %N) and the stable isotope pair ^13^C/^12^C (δ^13^C), which can be thought of as a proxy variable that reflect many physiological processes [4]. Samples were analysed by GC-IRMS using a Sercon ANCA elemental analyser, coupled to a Sercon 20-20 IRMS, located at the Environmental Stable Isotope Laboratory at the University of Leicester. Measurements were carried out in triplicates of each sample, resulting in precisions of ≤ 0.2‰ for stable isotope and ≤ 0.5% for C and N analysis. Outlier replicates were excluded from samples with lower levels of precision when necessary and not used to compute the average values of such samples. Isotopic values are reported in the standard δ–notation in per mil units (‰). δ^13^C values express the ^13^C content of a sample relative to the reference standard Vienna-Peedee belemnite (V-PDB), which has a ^13^C/^12^C abundance ratio of 1.1237 x 10^-2^ [5]. They were defined as:

δ^13^C (‰) = 1000[(*R*_sample_/*R*_V-PDB_)–1], where *R* = ^13^C/^12^C

where R_sample_ and R_V-PDB_ are the δ^13^C abundance ratios of the sample and reference standards, respectively. A lower sample value means that ^13^C is less abundant in the sample than in the reference standard.

It is important to note that only species that were sampled during the vegetation surveys at Upton and Woodwalton and for which we measured traits were included in trait analyses. We did not collect trait data for plant species that were not sampled at Upton and Woodwalton. Therefore, species that were present in the pollen data but not in the vegetation data were not included in any analysis (e.g., conifers).

**References**

1. Rodwell JS. British plant communities. Volume 1. Woodlands and scrub. Cambridge, UK: Cambridge University Press; 1991.

2. Pakeman RJ, Quested HM. Sampling plant functional traits: what proportion of the species need to be measured? Applied Vegetation Science. 2007;10(1):91-6.

3. Pérez-Harguindeguy N, Díaz S, Garnier E, Lavorel S, Poorter H, Jaureguiberry P, et al. New handbook for standardised measurement of plant functional traits worldwide. Aust J Bot. 2013;61(3):167-234.

4. Robinson D, Handley LL, Scrimgeour CM, Gordon DC, Forster BP, Ellis RP. Using stable isotope natural abundances (delta N-15 and delta C-13) to integrate the stress responses of wild barley (*Hordeum spontaneum* C. Koch.) genotypes. J Exp Bot. 2000;51(342):41-50.

5. Staddon PL. Carbon isotopes in functional soil ecology. Trends in Ecology & Evolution. 2004;19(3):148-54.
